# Screening of Single-Stranded DNA Aptamer Specific for Florfenicol and Application in Detection of Food Safety

**DOI:** 10.3390/bios12090701

**Published:** 2022-09-01

**Authors:** Minghui Shi, Ruobing Liu, Fuyuan Zhang, Bimal Chitrakar, Xianghong Wang

**Affiliations:** College of Food Science and Technology, Hebei Agricultural University, Baoding 071001, China

**Keywords:** florfenicol, SELEX, aptamer, AuNPs, molecular docking

## Abstract

In this work, the single-stranded DNA (ssDNA) aptamers specific to florfenicol (FF) and having a high binding affinity were prepared using the magnetic bead-based systematic evolution of ligands by the exponential enrichment technique (MB-SELEX). After 10 rounds of the MB-SELEX screening, aptamers that can simultaneously recognize FF and its metabolite florfenicol amine (FFA) were obtained. The aptamer with the lowest dissociation constant (K_d_) was truncated and optimized based on a secondary structure analysis. The optimal aptamer selected was Apt-14t, with a length of 43 nt, and its dissociation constant was 4.66 ± 0.75 nM, which was about 7 times higher than that of the full-length sequence. The potential binding sites and interactions with FF were demonstrated by molecular docking simulations. In addition, a colorimetric strategy for nanogold aptamers was constructed. The linear detection range of this method was 0.00128–500 ng/mL and the actual detection limit was 0.00128 ng/mL. Using this strategy to detect florfenicol in actual milk and eggs samples, the spiked recoveries were 88.9–123.1% and 84.0–112.2%, respectively, and the relative standard deviation was less than 5.6%, showing high accuracy.

## 1. Introduction

Throughout the animal farming system, the use of antibiotic medicines to control diseases is a common practice. However, an uncontrolled and haphazard use of antibiotics may cause an accumulation of their residues in animal-derived foods, such as milk and eggs [1]. The long-term use of such foods with antibiotic residues was reported to cause antibiotic-resistant infections in humans [2]. Therefore, it is of utmost importance to develop a convenient method to determine antibiotic content higher than the maximum residual limits (MRL) in animal products. Florfenicol (FF) is a broad-spectrum antibiotic from the amphenicol family, effective against both Gram-positive and Gram-negative bacteria; it is commonly used to cure infectious diseases in poultry, livestock, and aquaculture [3,4]. However, FF is not approved for human use because of its hematotoxicity and embryotoxicity, with the possibility of aplastic anemia. Florfenicol amine (FFA) is a dominant metabolite of FF, which exists in an animal body for the longest time after ingestion; the highest level is found in animal livers. Therefore, FFA is generally regarded as one of the indicative residues of florfenicol in animal food [5]. To detect the presence of FF residues in animal-derived foods, the MRLs are set as the sum of the FFA and FF [6,7]. The China National Standard (GB 31650-2019: National Food Safety Standard for Maximum Residue Limits of Veterinary Drugs in Foods) stipulates that the MRL of FF in different animal tissues is from 0.1 to 30.0 µg/g, while it is not permitted to have any residue in eggs and milk [8]. However, with the development of animal husbandry industries, the misuse of FF has led to its accumulation in livestock and poultry, resulting in a prominent food safety problem with a potential harm to people’s health. Therefore, it is crucial to develop sensitive and accurate detection methods for the regulation of FF residues in livestock and poultry products. At present, the detection of FF and FFA are mainly based on instrumental methods [9,10]. These methods have high sensitivity and accuracy, giving reliable results, but they need professional operators and require tedious pretreatments as well as being slow processes during on-site screening. Therefore, it is of great significance to establish a new detection technology that is easy to use and fast to detect FF in animal-derived foods.

Nucleic acid aptamers are single-stranded (ss) DNA or RNA that can bind specifically and with high affinity to target molecules and are obtained by a screening process called the systematic evolution of ligands by exponential enrichment (SELEX) [11]. The affinity of nucleic acid aptamers to the target molecule can come down to the level of mmol/L–pmol/L, which is similar to the level of antibodies. Moreover, they can be artificially synthesized in vitro; so, they are also called chemical antibodies [12,13]. Nucleic acid aptamers also have special advantages over antibodies, viz., aptamers can be synthesized in vitro at low cost; small batch-to-batch variation; and easy modification [14,15,16]. Moreover, the sensing strategy established with an aptamer as the biometric element highlights its advantages of rapid response and high sensitivity [17]. Aptamers have been widely used as a novel recognition compound in the detection and analysis of food contaminants, such as toxins [18], antibiotics [19], bacteria [20], and small organic compounds [21]. Currently, commonly used small-molecule targeted aptamer screening techniques include graphene oxide SELEX (GO-SELEX) [22,23], capture SELEX (C-SELEX) [24], and magnetic bead SELEX (MB-SELEX) [25]. Among them, MB-SELEX is the most commonly used SELEX screening method. The advantage of this method is that the magnetic beads have a spherical display surface, which is convenient for the binding of target substances and exposing the binding sites. The separation process using the magnetic field is simple, and fast, and can save target molecules. In addition, magnetic bead separation can be used for automated screening [26,27]. There are many types of surface modification for magnetic bead carriers, such as streptavidin-coated magnetic beads, amino-modified magnetic beads, carboxyl-modified magnetic beads, epoxy-activated magnetic beads, and tosyl-modified magnetic beads [28]. MB-SELEX has achieved great success in the screening of small-molecule aptamers, providing a simple and fast new method for the development of small-molecule nucleic acid aptamers.

At present, there are few studies on the screening of FF-specific aptamers. We used the MB-SELEX technique to screen ssDNA aptamers with a high affinity for FF and FFA; the selected aptamers were then truncated and then optimized to prepare a colorimetric strategy of gold nanoparticles (AuNPs). Finally, the gold nanoparticles-based colorimetric strategy was used to detect the FF residues in milk and eggs.

## 2. Materials and Methods

### 2.1. Raw Materials and Chemicals

Antibiotics, namely FF, FFA, chloramphenicol (CAP), and thiamphenicol (TAP), were purchased from Shanghai Yuanye Technology Biology Co., Ltd. (Shanghai, China). Graphene oxide (GO) was purchased from Guangzhou Wenrui Scientific Instrument Co., Ltd. (Guangzhou, China). Single-stranded DNA having 43 random nucleotides as the central sequence (5′-GCTGTGTGACTCCTGCAA-N43-GCAGCTGTATCTTGTCTCC-3′) and their primers, namely 5′-GCTGTGTGACTCCTGCAA-3′ as the forward primer and 5′-phosphate-GGAGACAAGATACAGCTGC-3′ as the 5′phosphorylated reverse one, were synthesized by Sangon Biotech Co., Ltd. (Shanghai, China) [29]. Tosyl magnetic beads were purchased from Biomag Biotech Co., Ltd. (Wuxi, China). All PCR materials (PCR buffer, ddH_2_O, and Mix) were ordered from Kangwei Century Biotech Co., Ltd. (Taizhou, China). Other chemicals, such as KCl, NaCl, and Tris-HCl, were purchased from Sinopharm Chemical Regent Co., Ltd. (Shanghai, China).

### 2.2. Immobilization of the FFA by Using Magnetic Beads

In the process of immobilization, FFA was covalently immobilized to the surface of magnetic beads activated by toluenesulfonyl (tosyl) groups. The tosyl-activated magnetic beads of 100 μL were washed successively with ice water and borate buffer (BBS) (0.1 M H_3_BO_3_, pH 9.5) and then separated by external magnetic field, the supernatant of which was discarded. The amino group from FFA was covalently attached by incubating the FFA (0.1 μmol) in BBS overnight at 37 °C under gentle rotation and tilting. After that, the supernatant was discarded to obtain FFA-coated magnetic beads (FMBs). The FMBs were washed with phosphate-buffered saline (PBS) buffer (1.5 M NaCl, 100 mM Na_2_HPO_4_·12H_2_O, 30 mM NaH_2_PO_4_·2H_2_O, pH 7.4) five times. Such beads were finally re-suspended in PBS buffer and stored at 4 °C for further use.

### 2.3. Screening of FF Aptamers by MB-SELEX

In vitro screening by MB-SELEX to isolate FF-specific aptamers. Before each round of SELEX, the FMBs were washed 5 times with the binding buffer (BB) (100 mM NaCl, 20 mM Tris-HCl, 2 mM MgCl_2_, 5 mM KCl, 1 mM CaCl_2_, 0.02% Tween 20, pH 7.6) and finally suspended in the 100 μL BB [29].

In the first round of SELEX, 1 nmol of random ssDNA library was dissolved in 100 μL BB as the initial library and was treated in a hot water bath at 90 °C for 10 min, followed by rapid cooling in an ice bath for 15 min. After that, the library was kept at room temperature for 7 min. The renatured library was quickly added to the washed FMBs for reaction at 37 °C and 160 rpm for 2 h. Washing was carried out with BB several times to remove the ssDNA that was not bound to the magnetic beads. Then, 100 μL elution buffer (EB; 10 mM EDTA-2Na, 3.5 M Urea, 50 mM Tris-HCl, 0.02% Tween 20, pH 8.0) was added into the magnetic beads; after vibration for 10 min at 80 °C, magnetic separation was applied to recover the ssDNA. The above steps were repeated 4 times to elute all traces of the combined ssDNA. The collected ssDNA library was further purified and concentrated by ethanol precipitation method. The purified ssDNA was redissolved in 30 μL distilled water and ultraviolet spectrophotometer (Unico Instruments Co., Ltd. Shanghai, China) was used to measure the concentration of ssDNA, combined with FFA. The best number of SELEX screening rounds was selected by calculating the recovery rate of ssDNA. Then, the purified ssDNA was amplified by polymerase chain reaction (PCR). The amplification process comprised following steps: first denaturation at 94 °C for 5 min; second denaturation at 94 °C for 30 s; then annealing at 51 °C for 30 s; followed by elongation at 72 °C for 30 s; and finally, elongation at 72 °C for 3 min. The PCR products were controlled by agarose gel electrophoresis to select the best PCR cycle without primer-dimer and non-specific products. The purified double-stranded DNA (dsDNA) was added to λ exonuclease, and the 5′ phosphorylated antisense chain of PCR product was removed by enzyme digestion to obtain ssDNA. The concentration of the obtained ssDNA was measured by ultraviolet spectrophotometer and used as the initial library in the next round of SELEX.

A total of 10 rounds of SELEX screening were carried out in this study. The amount of ssDNA library was gradually reduced as the number of SELEX screening rounds increased and the incubation time of the library and FFA was shortened. By increasing the screening pressure, the FF high-affinity aptamer was obtained. In addition, in the sixth round, negative screening was performed to improve the selection intensity. In the case of negative screening, the initial library from this round was first treated with a hot water bath at 90 °C, cooled to room temperature, and then reacted with 100 μL of unmodified FFA bare magnetic beads at 37 °C and 160 rpm for 2 h; then, the supernatant was taken and the ssDNA that was not combined with naked magnetic beads was added to FMBs. The reaction was carried out at 37 °C and 160 rpm for 2 h. Multiple washes with BB remove ssDNA that is not bound to the magnetic beads; then, the positive screening step was repeated.

### 2.4. Sequence Analysis and High-Throughput Sequencing

The final round of screening products was amplified by PCR and sent to Biotech Bioengineering (Shanghai, China) for high-throughput sequencing. The sequences with a high percentage of occurrences in the sequencing results were compared and analyzed by Mage 6 software and the secondary structures of the sequences were predicted by UNAFold (http://www.unafold.org/, accessed on 22 June 2021) [30]. Representative sequences with low free energy (ΔG) and stable structures were chosen from each family and synthesized with 5′-FAM markers for the next step in affinity identification. By analyzing the shared sequences and secondary structures between the candidate ssDNA, the most likely binding stem-loop positions were predicted for truncation and optimization.

### 2.5. Dissociation Constants (K_d_) Determination and Specificity Analysis of Candidate ssDNA

The 5′- FAM-labelled ssDNA sequence solution was heated and then rapidly cooled. The ssDNA sequences at different concentrations were incubated with a fixed concentration (1 M) of FF for 2 h, maintaining the total reaction volume of each mixture as 200 μL. A system without FF was also set up as the negative control. At the end of the reaction, GO was added in proportion to the mass of ssDNA (GO:ssDNA = 20:1), then incubated for 30 min away from light, and the fluorescence value of the supernatant was measured (excitation wavelength (Ex): 492 nm, emission wavelength (Em): 522 nm)). The dissociation constant K_d_ values were calculated according to the equation Y = B_max_ × X/(K_d_ + X) and analyzed by nonlinear fitting by using GraphPad Prism 6 software. In the equation, Y represents the fluorescence intensity of the supernatant; Bmax represents the number of maximum binding sites; and X represents the concentration of ssDNA. Data are expressed as mean ± standard deviation (SD) values; *n* = 3.

To assess the specificity of the aptamer, aptamers with high affinity were selected for analysis. After treatment with the same conditions as above, 100 nM aptamers were incubated with 1 µM of FF, FFA, CAP, and TAP, while BB was used as a blank control for incubation with aptamers, and then GO was added to adsorb unbound aptamers and the fluorescence values of the supernatant were measured (Ex = 492 nm, Em = 522 nm). The experiment was set up in three parallel replicates and treated under dark condition.

### 2.6. Molecular Docking Studies

The Lamarckian genetic algorithm of the AutoDock 4.2 software (Scripps Research, San Diego, CA, USA) was used for molecular docking of aptamer and FF. After the docking was completed, cluster analysis was performed on the multiple docking results and the binding conformation with the best docking score (that is, the smallest value) was selected from the optimal cluster, so as to determine the binding site between the target aptamer and FF as well as the type of interaction.

### 2.7. Detection of FF by AuNPs Colorimetry

The truncated optimal aptamer was used for the detection of FF by AuNPs colorimetry, and its practicability was verified.

AuNPs was prepared by the sodium citrate reduction method. A total of 1 mL of 1% chloroauric acid solution was mixed with 99 mL ultrapure water, which was subjected to boil and then 1 mL of 1% trisodium citrate was added. It was observed that the color of the solution changed from colorless to gray, then to black, and finally to orange. Heating was stopped and the solution was cooled down to room temperature by continuous stirring. The solution was packed in a brown reagent bottle to obtain AuNPs, which were protected from light and stored at 4 °C.

The truncated aptamer (200 nM) was reacted with 50 μL AuNPs in the dark for 30 min and the total system was 200 μL. A series of concentrations of FF were added to the system and the reaction was performed at 37 °C for 1 h in the dark. After the reaction, 10 μL of NaCl (1 M) was added to the system, and the reaction was performed for 7 min. The absorbance was measured by a microplate reader (Beijing Boao Hengxin Biotechnology Co., Ltd., Beijing, China) at wavelength values of 650 and 520 nm (A650/A520). In order to evaluate the specificity of this method, under the same conditions as above, the aptamer was reacted with AuNPs; excess CAP and TAP were added; and the absorbance value (A650/A520) was measured by a microplate reader.

In order to verify the practicability of colloidal gold colorimetry to detect FF, this method was used to detect FF in real samples of milk and eggs. The concentrations of FF in milk and egg samples were 0.1, 1, and 5 ng/mL, and then the samples were pre-treated: 1 mL of milk (egg) was homogenized, 3 mL of ethyl acetate was added and mixed evenly, ultrasonicated for 5 min, centrifuged at 8000 r/min for 10 min. After centrifugation, 1 mL of the supernatant was taken and concentrated by nitrogen blowing at 60 °C. The concentrated sample was added to 1 mL of ultrapure water to dissolve, and after mixing, sonicated for 5 min, and then 1 mL of n-hexane was added. Ultrasonic for 5 min, then centrifuged at 8000 r/min for 10 min, remove the upper n-hexane phase, and analyze the obtained lower aqueous phase. After removing the influence of the matrix, the egg samples were diluted 10 times and the milk samples were diluted 2 times. After the aptamer was reacted with 50 μL AuNPs in the dark for 30 min, the spiked egg or milk sample was added, and the reaction was carried out at 37 °C in the dark for 1 h. After the reaction, 10 μL of NaCl (1 M) was added to the system. After 7 min, a microplate reader was used to determine the absorbance value (A650/A520).

## 3. Results and Discussion

### 3.1. Immobilization of Targets on Magnetic Beads

**Figure 1:**
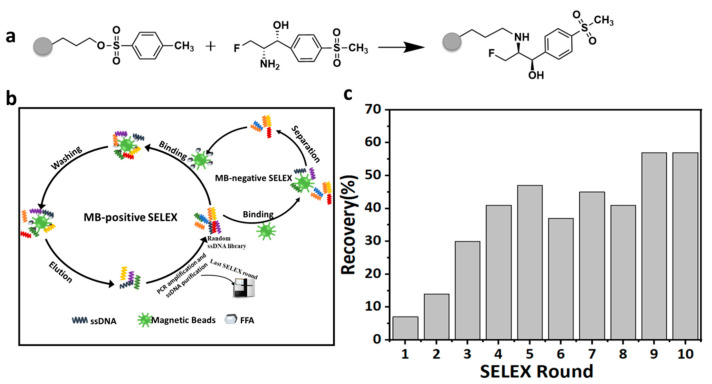
(**a**) The scheme of the covalent coupling of florfenicol amine on tosyl-activated magnetic beads. (**b**) Schematic diagram of MB-SELEX. (**c**) Recovery of ssDNA in the solution in each round of selection.

Aptamers with a high affinity for FF were selected from random libraries using tosyl-activated magnetic beads. FFA is a metabolite of FF, and the FF amide bond breaks to form the FFA. Because the FFA has only one amine (NH_2_) functional group, tosyl is a leaving group that can be replaced by a primary amine group. According to this feature, the toluenesulfonyl-activated beads were selected as the target molecule-immobilized materials. The FFA was immobilized on magnetic beads to obtain the FMBs (Figure 1a). Moreover, an excess of the FFA was used to ensure that all the binding sites of the magnetic beads were occupied by the FFA.

Fourier transform infrared spectroscopy (FTIR) was used to confirm that the target molecule was immobilized on the magnetic beads. Figure S1a is the FTIR spectrum of the FFA, where the strong peak at 1100 cm^−1^ represents the C-F vibration. In addition, two peaks at 3300–3500 cm^−1^ are related to the N-H stretching vibrations of the primary amines. As shown in the FTIR spectrum (Figure S1b), the tosyl-activated magnetic beads had a distinct peak around 1500 cm^−1^, corresponding to the C=C aromatic vibration, which was one of the characteristics of the tosyl-activated beads. As shown by the FTIR spectrum (Figure S1c), the FFA was bound to the tosyl-activated bead surface. The presence of a peak near 1100 cm^−1^ corresponded to the C-F stretch, indicating the presence of FFA in the conjugated structure. In addition, the disappearance of the primary amine peak around 3300–3500 cm^−1^ and the appearance of the peak corresponding to the secondary amine in the target-coated beads confirmed the successful coupling of the FFA through the amine group.

### 3.2. Selection of Aptamer In Vitro

The screening of the FF-specific aptamers was accomplished by the MB-SELEX procedure. In each round of the screening, the library was first incubated and combined with FMBs; the ssDNA not bound to the target was removed by magnetic separation; the ssDNA specifically bound to the target was separated from the magnetic beads by elution; and the obtained ssDNA was subjected to PCR amplification. After the purification of the PCR product, the dsDNA was cut into the ssDNA by the λ exonuclease [31], and the resulting ssDNA library was used as the initial library for the next round. During the negative screening, the library was incubated with unmodified FFA bare magnetic beads for binding and then magnetically separated to obtain the ssDNA that was not bound to the bare magnetic beads. After incubating the resulting ssDNA with FMBs, the positive screening step was repeated (Figure 1b). In order to obtain ssDNA with a higher affinity for the target FF, the screening pressure was gradually increased as the number of SELEX screening rounds increased. By gradually reducing the amount of the ssDNA library, the incubation time between the library and FFA was gradually shortened from 120 to 90 min and the negative screening was performed in the sixth round to improve the selection strength (Table S1). With the increase in the number of screening rounds, the recovery rate of the ssDNA gradually increased. To a certain extent, the recovery rate of the ssDNA dropped in the 6th, 8th, and 10th rounds of screening. Such consequences could be attributed that the negative screening removed some false-positive sequences. Finally, the recovery rate of the ssDNA reached saturation in the tenth round (Figure 1c). It showed that after ten rounds of screening, the ssDNA with a higher affinity for FF was gradually enriched, while the ssDNA with a relatively lower affinity was gradually eliminated. The ssDNA from the 10th round of screening was PCR amplified and cloned for sequencing.

### 3.3. Characterization of Specific Ligands

After 10 rounds of screening and enrichment, the library was sequenced with high throughput. The 20 sequences with the most repeats were evaluated using the Mage 6 software (Auckland, New Zealand). According to their homology and phylogenetic tree, all the selected aptamers were divided into three families, and the secondary structure of the sequence was predicted by the UNAFold structure software. The representative aptamers were rich in the conservative motifs “GGT” and “TGG” (shown in bold letters), which might be important during recognition and binding to the target. Finally, four aptamers (indicated by standard red) of Apt-1, Apt-14, Apt-15, and Apt-20 with low free energy and a stable structure were selected (Table S2). In the secondary structure of the four aptamers simulated and predicted by the UNAFold structure software (Figure S2), the conservative pattern “GGT” was usually located in the ring region. These structural characteristics might be related to the specific binding of the aptamers to the FF.

The smaller the K_d_ value, the higher the affinity of the aptamer to the target. According to this feature, the K_d_ values of the candidate aptamers were measured by the fluorescence method to evaluate their affinity with the FF. Four aptamers, namely Apt-1, Apt-14, Apt-15, and Apt-20, were chemically synthesized and labeled with FAM at the 5′ end.

**Figure 2:**
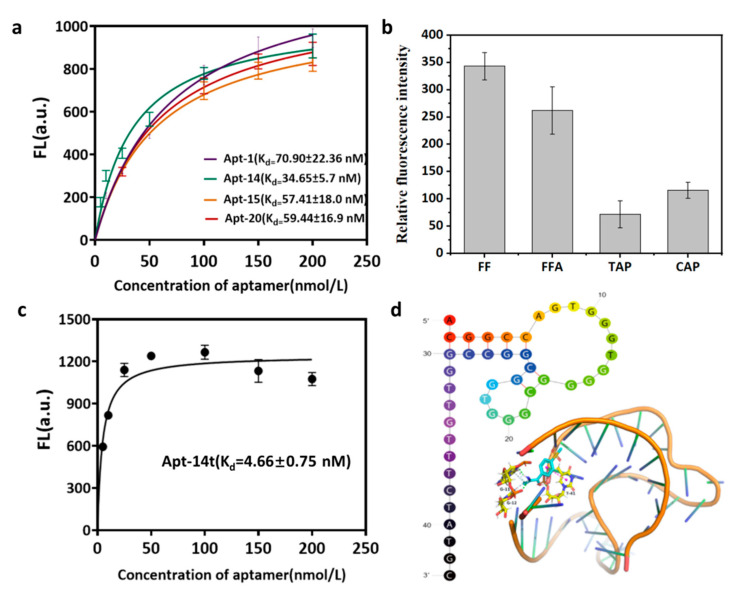
(**a**) The saturation curves and K_d_ values of Apt-1, Apt-14, Apt-15, and Apt-20. (**b**) Relative fluorescence intensity of Apt-14t in the presence of florfenicol, florfenicol amine, chloramphenicol, and thiamphenicol. (**c**) The nonlinear regression analysis of Apt-14t (0–200 nmol L^−1^) for evaluation of K_d_ values. (**d**) Secondary structure of the truncated aptamers and molecular docking of Apt-14t and FF.

The FAM-labeled aptamers were adsorbed on the surface of the GO by π stacking and a hydrophobic interaction, and the fluorescence of the aptamers was quenched due to the fluorescence resonance energy transfer (FRET). The presence of the FF resulted in a competitive binding of the FF to the aptamer, which caused its desorption from the GO, ultimately inhibiting the FRET and restoring the fluorescence of the aptamers. By fixing the FF concentration and the gradient aptamer concentration, the nonlinear fitting curve was drawn by the GraphPadPrism6 software, and the dissociation constant (K_d_) was calculated according to the equation Y = B_max_ × X/(K_d_ + X). Among them, the K_d_ value of Apt-14 was the lowest (34.65 ± 5.7 nM), and the affinity with FF was the best (Figure 2a). In order to reduce the cost and to obtain the aptamer with a high affinity for the FF, Apt-14 was optimized. The truncation was carried out in a semi-rational manner, that is, the truncation scheme was based on both the structural prediction and affinity analysis [32]. The truncation will be discarded if the affinity of the truncated aptamer decreases. Based on the secondary structure of Apt-14, the middle hairpin structure was intercepted to obtain the aptamer Apt-14t, whose sequence is “ACGGCCAGTGGGTGGGGCGGGTGGCGGCCGGTTGTTTCTATGC”, and the length is 43 nt. The secondary structure of Apt-14t was simulated and predicted by the UNAFold structure software. The truncated aptamer structure was stable and showed the hairpin structure. The K_d_ value of the aptamer was determined by the fluorescence method to evaluate its affinity with the FF. The K_d_ value of the aptamer was 4.66 ± 0.75 nM by the nonlinear fitting obtained from the GraphPadPrism6 software, which was about 7 times higher than that of the full-length sequence (Figure 2b). The affinity of Apt-14t to the target was significantly enhanced, which might be attributed to the deletion of redundant sequences. Sadeghi and coworkers screened out the FF aptamer with a sequence of “GCTGTGTGACTCCTGCAAGGTCCATTCAAGTCGTAGGTTTGCCTTCAGCCTCAACGCTTACGCAGCTGTATCTTGTCTCC” by using the FluMag-SELEX technique with magnetic beads, having its K_d_ value as 52.78 nM [29]; Xiao and coworkers adopted the GO-SELEX technology to screen out the nucleic acid of the FF aptamer; the sequence was “GCCCACAGTGTTGCGGGAATGATTATCCGCCGAGGGGTGG” and its K_d_ value was 11.811 ± 4.030 nM [33]. Compared with the above two sequences, Apt-14t screened by the MB-SELEX in this paper had the highest affinity for the FF.

The specificity of Apt-14t was then evaluated. With the sample without any antibiotics as the control group, the relative fluorescence intensities of the solutions in the presence of the selected antibiotics and the FF were compared. Because the FFA is a metabolite of the FF, the aptamer can also bind the FF well. The relative fluorescence intensities obtained in the presence of the FF and FFA were much higher than those of other antibiotics, with the negligible binding of antibiotics from other families (Figure 2c). This indicated that the aptamer had a higher specificity.

### 3.4. Molecular Docking

The Lamarckian genetic algorithm of the AutoDock4.2 software was used to dock the FF and Apt-14t (Figure 2d). The results showed that the FF was bound to G11, G12, and T41 of the aptamer through a hydrophobic interaction and hydrogen bonding.

According to the secondary structure analysis and binding site prediction, the length of the aptamer was reduced after deleting the redundant sequence, and the affinity of the truncated aptamer to the target was increased by 7 times, compared with the full-length sequence. Therefore, we believed that the selected optimal aptamers laid a solid foundation for their further application in the detection of FF by gold colorimetry.

### 3.5. Detection of FF by AuNPs Colorimetry

**Figure 3:**
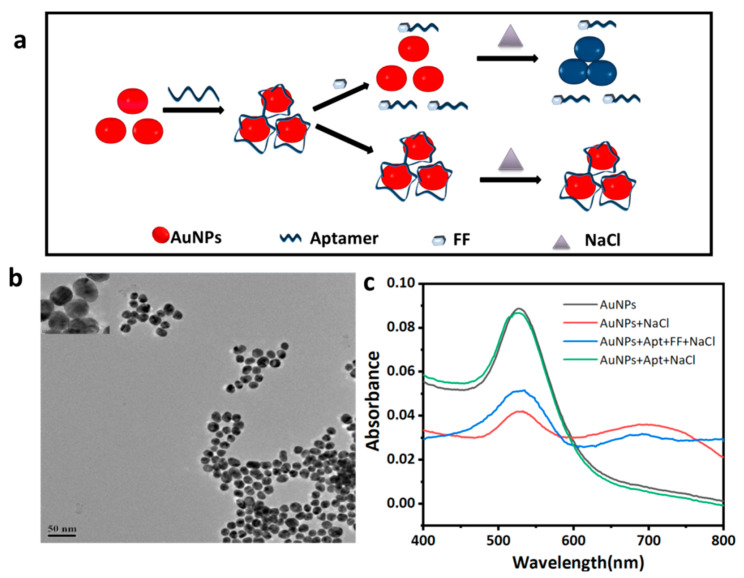
(**a**) Schematic diagram of the aptamer and nanogold assay FF. (**b**) Transmission electron microscope scan of AuNPs. (**c**) UV—vis absorption spectra of different systems.

The AuNPs aggregate, after reacting with NaCl, changes the color from red to blue. The ssDNA aptamers are adsorbed on the surface of the AuNPs through the van der Waals forces and hydrophobic interactions between N and O on the nucleic acid bases, protecting the AuNPs from salt-induced aggregation and making the AuNPs in a dispersed state and red in color. In the presence of the FF, the aptamer specifically bound to the FF, causing the AuNPs to lose protection, aggregate under the action of salt solution, the color changes from red to blue, while the absorbance value increased (Figure 3a). Changes in the absorbance value of the solution at 520 nm (A520) and 650 nm (A650) was observed by visible spectrophotometry. The rule of thumb is that the larger the value of A650/A520, the higher the florfenicol content in the sample.

#### 3.5.1. AuNP Characterization

Commonly used colloidal gold preparation methods include the trisodium citrate reduction method, tannic acid reduction method, white phosphorus reduction method, etc. The particle size of colloidal gold varies with the amount of reducing agent added. In this experiment, the trisodium citrate reduction method was used to prepare the colloidal gold. The whole method is simple and inexpensive, which can quickly prepare colloidal gold with different particle sizes. In order to prove that the AuNPs were successfully prepared and the particle size and micro-morphology of the AuNPs were suitable for the construction of the colorimetric sensors, the prepared AuNPs were scanned through a transmission electron microscope (Figure 3b). The prepared AuNPs were observed to have regular spheres of a uniform size (about 17 nm in diameter) with good dispersibility that can be used for the construction of nanogold colorimetric strategies.

The principle of the colloidal gold colorimetric detection of the FF was verified (the whole reaction was carried out in the binding buffer system, and the pH value was 7.6). In the UV spectrum (Figure 3c), compared with the AuNPs solution system without the NaCl, the absorption peak of the system with the NaCl was found to decrease at around 525 nm, while a significant absorption peak appeared at around 700 nm. It showed that, under the action of NaCl, the AuNPs changed from a dispersed state to an aggregated state. The UV absorption spectrum of the Apt-AuNPs with NaCl was similar to that without NaCl, which proved that the aptamer adsorbed on the surface of the AuNPs was able to play a certain protective role and made the AuNPs remain dispersed in the presence of NaCl. When the FF was added, the absorption peak of the system at 520 nm decreased, and a new absorption peak appeared at 700 nm, indicating that the FF bound specifically to the aptamer, which made the AuNPs lose their protection and begin to reunite after reacting with the NaCl. The above results demonstrated that the synthesized colloidal gold can be applied in this method.

#### 3.5.2. Condition Optimization

**Figure 4:**
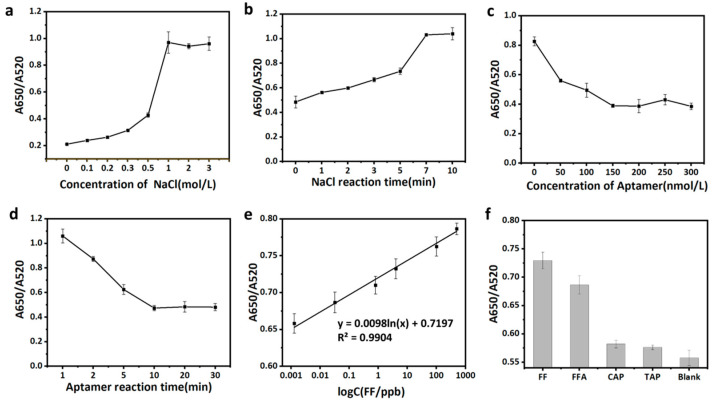
Optimization of AuNPs colorimetric conditions: (**a**) Optimization of NaCl concentration. (**b**) Optimization of NaCl reaction time. (**c**) Optimization of aptamer concentration. (**d**) Optimization of reaction time between aptamer and AuNPs. (**e**) The linear relationship between absorbance and the concentration of FF in standard solution. A650 is the absorbance value at 650 nm and A520 is the absorbance value at 520 nm. (**f**) Relative fluorescence intensity of Apt-14t in the presence of florfenicol, florfenicol amine, chloramphenicol, and thiamphenicol.

In order to obtain the best performance, the reaction conditions of the nanogold colorimetric sensor were optimized. The optimization was conducted for the concentration of NaCl, the reaction time after adding NaCl to the system, the concentration of the aptamer, and the incubation time of the aptamer and gold nanoparticles. Among them, the concentration of the NaCl and the reaction time of the NaCl had an important influence on the agglomeration of the AuNPs. The ratio of A650/A520 in the AuNPs system was also optimized. With the increase in the NaCl concentration and reaction time, the value of A650/A520 increased gradually. When the concentration of the NaCl was higher than 1 M and the reaction time was longer than 7 min, it tended to be stable (Figure 4a,b). The aptamer concentration had an important effect on the sensitivity and stability of the sensor. When the aptamer concentration was higher than 200 nM, the value of A650/A520 tended to be stable (Figure 4c). The incubation time of the aptamer and gold nanoparticles affected the aggregation of the AuNPs and the ratio of A650/A520 tended to be stable when the incubation time was more than 10 min (Figure 4d). Based on the above optimization, the NaCl concentration, the reaction time after adding the NaCl to the system, the concentration of the aptamer, and the incubation time of the aptamer and gold nanoparticles were set as 1 M, 7 min, 200 nM, and 10 min, respectively.

#### 3.5.3. Colorimetric Method Performance Verification

Under the optimum conditions, the relationship between the absorbance of the nanogold colorimetric sensor and the concentration of the FF was studied. With the increase in the FF concentration in the sample, the value of A650/A520 increased gradually (Figure 4e). Within the range of 0.00128–500 ng/mL, there was a good linear relationship between the value of A650/A520 and the concentration of the FF. The linear regression equation is y = 0.0098 ln(x) + 0.7197 (R^2^ = 0.9904), where y represents the A650/A520 value and x represents the concentration of the FF. The actual detection limit of the sensor was 0.00128 ng/mL.

In order to evaluate the selectivity of the nanogold colorimetric strategy, the absorbance value response of the nanogold colorimetric method to the FF, FFA, and the structural analogs of the CAP and TAP was determined under similar experimental conditions. The A650/A520 values in the presence of the FF or FFA were higher than other antibiotics, indicating better specificity of the method (Figure 4f). Compared with the other reported detection techniques, this newly constructed aptamer sensor was more economical and faster. In addition, the sensor had a lower detection limit (LOD) and had good feasibility for the detection of FF in actual milk and egg samples (Table 1).

### 3.6. Detection of FF in Milk and Egg Samples

In order to verify the feasibility of the Apt-14t-based nanogold colorimetry in the analysis of actual samples, it was applied to the detection of FF in spiked milk and egg samples. In the milk samples, the detection method showed a good linear relationship between the absorbance value and the FF concentration in the range of 0.07825–20 ng/mL. The linear regression equation is y = 0.0404 ln(x) + 0.6246 and the lowest practical detection limit measured is 0.07825 ng/mL with an R² value of 0.989 (Figure S3a). In the egg samples, the detection method showed a good linear relationship between the absorbance value and the FF concentration in the range of 0.02–62.5 ng/mL. The linear regression equation is y = 0.0253 ln(x) + 0.5557, and the lowest practical detection limit is measured as 0.02 ng/mL with an R² value of 0.992 (Figure S3b). The spiked recoveries of the florfenicol in the milk and eggs were 88.9 to 123.1% and 84.0 to 112.2%, respectively, with a relative standard deviation of less than 5.6%, showing a high accuracy (Table 2). These results demonstrated that the Apt-14t-based nanogold colorimetry had desirable properties for the determination of FF in real samples.

## 4. Discussion

In this study, the aptamer with the specific recognition of the FF and FFA was successfully selected by 10 rounds of MB-SELEX and its truncation was optimized to obtain Apt-14t, which improved the affinity by 7 times, compared with the full-length sequence. The molecular docking indicated that the stem-loop of Apt-14t was a more likely site to bind with FF. A colorimetric strategy for the AuNPs was established using Apt-14t and spiked for the recovery of the milk and egg samples. Compared with other reports, the limit of detection (LOD) of the AuNPs colorimetric method was the best, and the lower limit of detection was 0.00128 ng/mL. Additionally, high sensitivity, satisfactory recovery, and RSD were obtained in the actual sample detection.

## Figures and Tables

**Table 1:** Comparison of this method with other detection methods.

Methods	Detection Range (ng/mL)	LOD (ng/mL)	Reference
icELISA	0.31–5.61	0.12	[34]
Colloidal Gold Lateral Flow Immunoassay	0.1–1.5	0.08	[35]
UPLC	12.5–1000	12	[36]
Electrochemiluminescence immunoassay	0.0001–100	3.1 × 10^−^^5^	[37]
FMs lateral flow assay	1.25–80	1.9	[38]
HPLC-MS-MS	0.1–500	2.9 × 10^−3^	[39]
Nanogold Colorimetry	0.00128–500	1.28 × 10^−3^	This work

**Table 2:** Detection of FF in milk and egg samples.

Sample	Spiked (ng/mL)	Found (ng/mL)	Recovery (%)	RSD (%)
	0.1	0.11	112.2	5.6
Milk	1	1.23	123.1	2.3
	5	4.45	88.9	2.7
	0.1	0.09	92.4	5.1
Egg	1	0.84	84.0	1.6
	5	5.61	112.2	4.0

## Data Availability

Not applicable.

## Supplementary Materials

The following supporting information can be downloaded at: https://www.mdpi.com/article/10.3390/bios12090701/s1, Figure S1: Infrared spectra of (a) florfenicol amine, (b) toluenesulfonyl-activated beads, and (c) florfenicol amine-coated magnetic beads from 250 to 4000 cm^−1^; Figure S2: The secondary structures of four selected aptamers; Figure S3: Standard curve for the determination of FF using the developed aptasensor upon addition of different concentrations of FF in (a) milk and (b) egg; Table S1: MB-SELEX screening criteria; Table S2: The 43 nt random regions of the screened aptamers and their classification.
