# Supplementary material for: Screening of Single-Stranded DNA Aptamer Specific for Florfenicol and Application in Detection of Food Safety
Source: Biosensors (Basel). 2022 Sep 1;12(9):701. doi: 10.3390/bios12090701 (PMC9496042; doi:10.3390/bios12090701)
Supplement: Supplementary file 1 [file biosensors-12-00701-s001.zip › biosensors-1872803-supplementary.pdf]

Supplementary Materials:

Screening of Single-Stranded DNA Aptamer Specific for Florfenicol and Application in Detection of Food Safety

Minghui Shi †, Ruobing Liu †, Fuyuan Zhang, Bimal Chitrakar and Xianghong Wang *

College of Food Science and Technology, Hebei Agricultural University, Baoding 071001, China
* Correspondence: wangshipin2017@163.com
† These authors contributed equally to this work.

Figure. S1. Infrared spectra of (a) Florfenicol amine (b) Toluenesulfonyl activated beads, and (c) Florfenicol amine-coated magnetic beads from 250 to 4000 cm^{-1}.
Figure. S2. The secondary structures of four selected aptamers.
Figure. S3. Standard curve for the determination of FF using the developed aptasensor upon addition of different concentrations of FF in (a) Milk and (b) Egg.
Table S1. MB-SELEX screening criteria.
Table S2. 43 nt random regions of the screened aptamers and their classification.

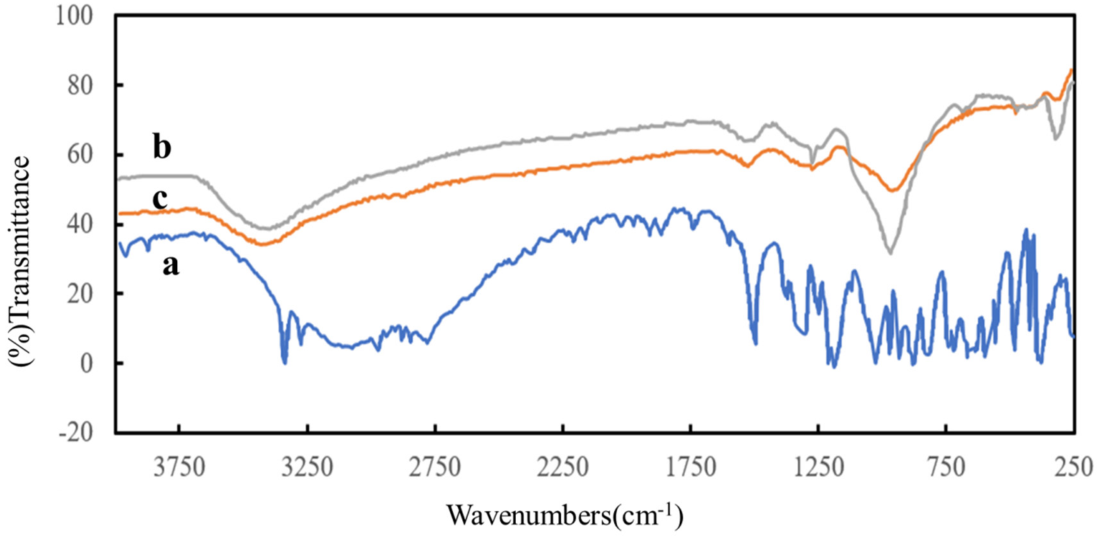

Figure S1. Infrared spectra of (a) Florfenicol amine (b) Toluenesulfonyl activated beads, and (c) Florfenicol amine-coated magnetic beads from 250 to 4000 cm^{-1}.

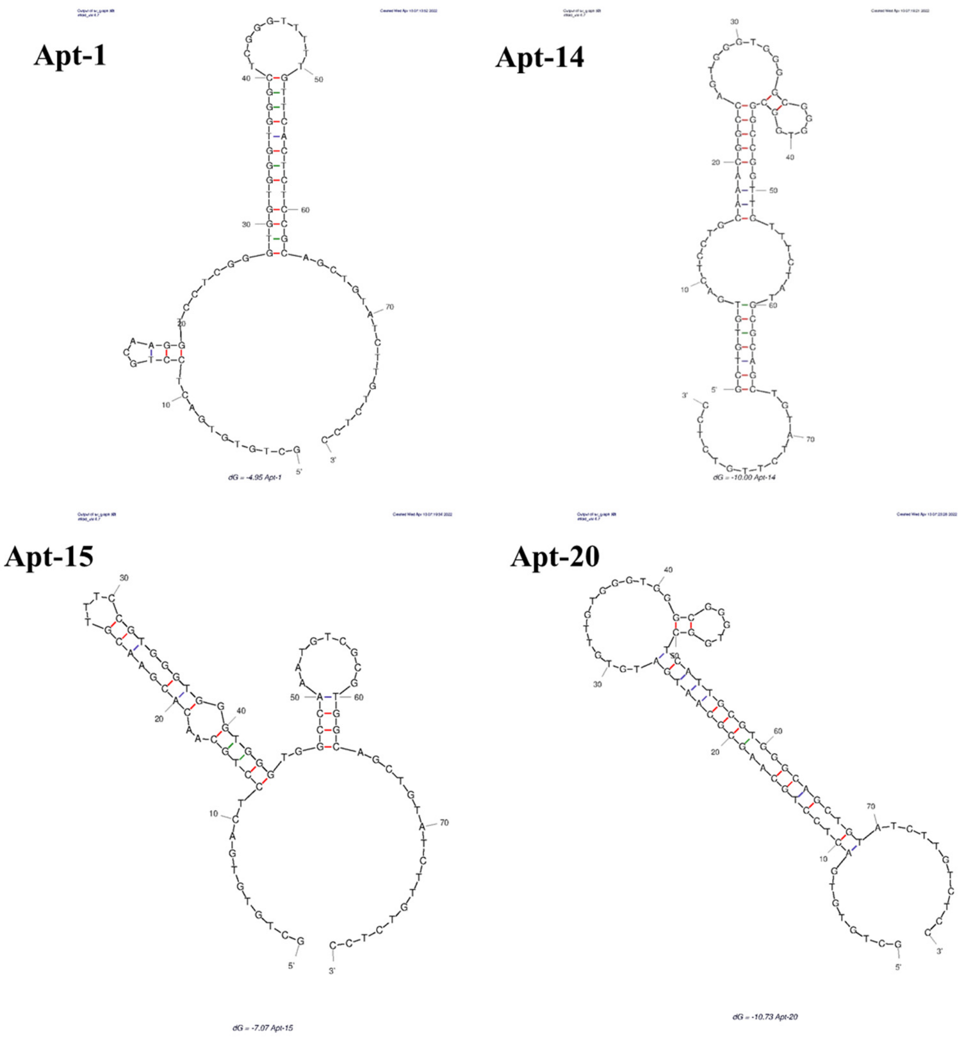

Figure S2. The secondary structures of four selected aptamers.

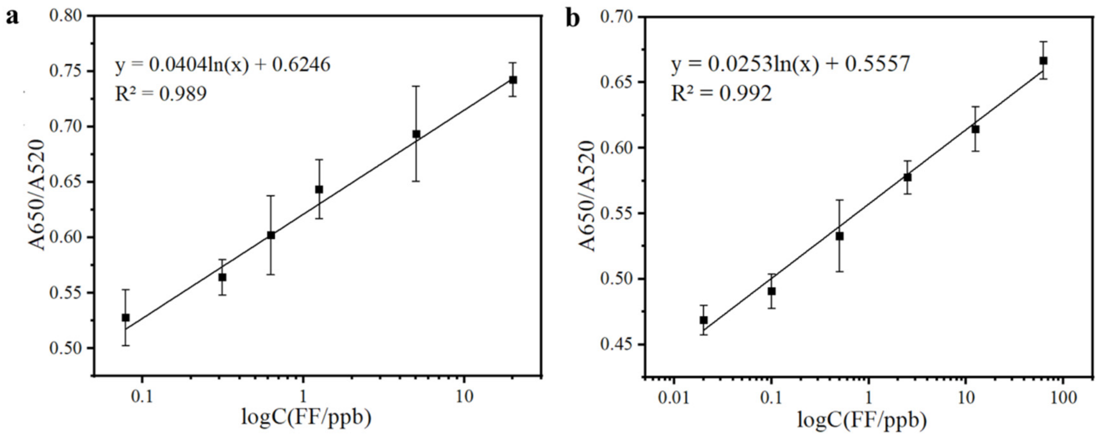

Figure S3. Standard curve for the determination of FF using the developed aptasensor upon addition of different concentrations of FF in (a) Milk and (b) Egg.

Table S1. MB-SELEX screening criteria.

SELEX Round	ssDNA Added (pmol)	Time (min)	Negative SELEX
1	1000	120	-
2	366	120	-
3	394	120	-
4	10	100	-
5	13.3	100	-
6	303	100	Yes
7	235	90	-
8	220	90	Yes
9	292	90	-
10	189	90	Yes

Table S2. 43 nt random regions of the screened aptamers and their classification.

Family	Aptamer	Intermediate random sequence（5′-3′）	ΔG（kcal/mol）
	Apt-1	GGTCCTCGGGTGGTGGGTGGGCTCGGGTTTTTGTTCACTCTCC	-4.95
	Apt-5	CAGAATATG**GGTGGT**G**GGT**GGGCTCGGTCTTTCCTCTATG**TGG**	-2.21
	Apt-7	GCGTCCGTACGT**TGG**GGGCG**GGT**GGG**TGG**ATAGTCTTCGCCTT	-6.93
	Apt-8	CCG**GGTGG**GG**GGTGG**GCTCGGACCTTGTGTATTCCGTTTACTC	-3.82
I	Apt-13	GGTCCTCGGGT**GGTGG**GTGGGCTCGG**GGT**TTTGTTCACTCTCC	-4.95
	Apt-14	ACGGCCAGTG**GGTGG**GGCG**GGTGG**CGGCC**GGT**TGTTTCTATGC	-10.00
	Apt-16	CAG**TGG**GTG**GGTGG**GTGGAAGTCGGCGGAATTAATTTTGGCAC	-4.86
	Apt-18	GGTCCTCGGGG**GGT**G**GGTGG**GCTCGGGTTTTTGTTCACTCTCC	-5.39
	Apt-19	CGGTG**TGG**GCGGGTG**GGTGG**ATACGTTGCAGCGTATGGTACCC	-9.59
	Apt-3	CCCGGGGGGGGACTCTAAAAGGG**GGT**AAATGTAT**TGG**GGGTGG	-4.18
	Apt-4	TCT**GGTGG**GTGGGGCTGGGGGTGGTCTAGTTCCGCGTGCGCTT	-5.76
II	Apt-6	CATGAGGATAGCC**GGTGG**GCGGGTGGGAGGAGGCAGCCCTTGT	-8.42
	Apt-12	AATCCCGGGTGGAG**GGTGG**GCTCGGTATATAGTACATTCTTGG	-3.06
	Apt-20	GCGCAATGATGTGTTGT**GGGTGG**GCG**GGTGG**CTCATTGCGTGG	-10.73
	Apt-2	TATGG**GGTGG**TGGGCGGGCTTC**GGT**CATTGTGTGTTATCTTTC	-3.53
	Apt-9	TG**GGTGG**GGGGGGGTGGATCCGTTAGTAATGGGGGCTGGCTAG	-6.48
	Apt-10	TACACGTTGGGC**GGTGG**GTGGGTCTAGCCATGTCTTGCTTATA	-5.16
III	Apt-11	GGACG**GGTGG**TGGGTGGGCT**TGG**ACATAGTAGACATTTGGTTT	-3.69
	Apt-15	CACGAACGTTTCCGTGGGTGGGTGGGTGGCCAAATGTCGCGTG	-7.07
	Apt-17	CC**GGT**GT**GGGTGG**GTG**GGTGG**TTATGC**GGT**TGAAGTCCCGATG	-6.17
